# Supplementary material for: Tuna behaviour at anchored FADs inferred from Local Ecological Knowledge (LEK) of pole-and-line tuna fishers in the Maldives
Source: PLoS One. 2021 Jul 29;16(7):e0254617. doi: 10.1371/journal.pone.0254617 (PMC8321119; doi:10.1371/journal.pone.0254617)
Supplement: S1 Appendix — (DOCX) [file pone.0254617.s001.docx]

**S1 Appendix**

**Local Ecological Knowledge – Fisher interview questionnaire**

1. During which season (monsoon) are there more fish (any size) around different types of schools?

|  | Skipjack tuna | | | | | | | | | | | | Yellowfin tuna | | | | | | | | | | | |
| --- | --- | --- | --- | --- | --- | --- | --- | --- | --- | --- | --- | --- | --- | --- | --- | --- | --- | --- | --- | --- | --- | --- | --- | --- |
| **School Type** | **J** | **F** | **M** | **A** | **M** | **J** | **J** | **A** | **S** | **O** | **N** | **D** | **J** | **F** | **M** | **A** | **M** | **J** | **J** | **A** | **S** | **O** | **N** | **D** |
| AFAD |  |  |  |  |  |  |  |  |  |  |  |  |  |  |  |  |  |  |  |  |  |  |  |  |
| Seamount |  |  |  |  |  |  |  |  |  |  |  |  |  |  |  |  |  |  |  |  |  |  |  |  |
| Free school |  |  |  |  |  |  |  |  |  |  |  |  |  |  |  |  |  |  |  |  |  |  |  |  |
| DFAD |  |  |  |  |  |  |  |  |  |  |  |  |  |  |  |  |  |  |  |  |  |  |  |  |
| Log school |  |  |  |  |  |  |  |  |  |  |  |  |  |  |  |  |  |  |  |  |  |  |  |  |

1. Is there any variation in abundance of fish around AFADs on the east and west of Maldives? (Are there more fish on the east during northeast monsoon? Are there more fish on the west during southwest monsoon?)
2. Are there any AFADs that generally attract less fish?
3. Are there any AFADs that generally attract more fish?
4. When AFADs are close by (adjacent) do more fish appear on one AFAD than other or are they equal?
5. When a FAD aggregates fish how many days is the aggregation around?

*Options provided:* ***<3days, 3 to 6 days, 7 to 10 days, >10days***

1. Which are the most important factors that help the formation of fish aggregation at AFADs?

*Options provided:* ***current, temperature, turbidity, presence of prey, attractants attached, sea state.***

1. Which are the most important factors that explains why fish leaves the AFADs?

*Options provided:* ***current, temperature, turbidity, absence of prey, absence of attractants, stormy seas, presence of large predators, large size of the aggregations.***

1. What is the distance that you consider that the fish is still associated to the AFAD?

*Options provided:* ***0 to 2 miles, 0 to 5 miles, >5 miles***

1. Does time of the day influence the behavior of tuna at the AFADs?

***Options provided: horizontal distance from AFAD, vertical distance from AFAD and catchability***

1. Do you think there are multiple schools of fish at the AFADs?
