# Supplementary material for: Tuna behaviour at anchored FADs inferred from Local Ecological Knowledge (LEK) of pole-and-line tuna fishers in the Maldives
Source: PLoS One. 2021 Jul 29;16(7):e0254617. doi: 10.1371/journal.pone.0254617 (PMC8321119; doi:10.1371/journal.pone.0254617)
Supplement: Attachment — Submitted filename: LEKMaldives_ARJauharee_EditorComments.docx [file pone.0254617.s004.docx]

Tuna behaviour around anchored FADs inferred from Local Ecological Knowledge (LEK) of pole-and-line tuna fishers in the Maldives

A Riyaz Jauharee^1,2*^, Manuela Capello^2^, Monique Simier^2^, Fabien Forget^2^, M Shiham Adam^3^, Laurent Dagorn^2^

^1^Maldives Marine Research Institute, Ministry of Fisheries, Marine Resources and Agriculture.

^2^MARBEC, University of Montpellier, CNRS, Ifremer, IRD, Sète, France

^3^International Pole and Line Foundation, 1 London Street, Reading, RG1 4QW, United Kingdom.

* Corresponding author

E-mail: arjauharee@gmail.com

Funding: This study was fully funded by International Pole & Line Foundation (IPNLF)

Acknowledgements: At foremost authors thank the Maldivian tuna fishers who contributed to this study by sharing valuable information on tuna fishing around aFADs. In addition, we also acknowledge International Pole-and-line Foundation (IPNLF) for funding this study. Maldives Marine Research Institute of the Ministry of Fisheries, Marine Resources and Agriculture (MoFMRA) was also instrumental in facilitating the field work.

Abstract

The Maldives tuna fishery landings in 2018 was 148, 000t and accounts for nearly a quarter of the global pole-and-line tuna catch. This fishery partly relies on a network of 55 anchored fish aggregating devices (aFADs) deployed across the archipelago. Although the aFAD fishery has existed for 35 years and about 30 to 50% of the total tuna catch is harvested from aFAD schools, knowledge on the behaviour of tuna in this low density aFAD array is still poor, precluding from developing evidence-based fishery management. In this study, local ecological knowledge (LEK) of fishers was used to improve the understanding on tuna behaviour, through personal interviews of 54 pole-and-line fishers from different parts of the archipelago. Interview results suggest that during the northeast monsoon there are more tuna on the east, while in the southwest monsoon there are more tuna on the west of the Maldives. Most fishers believed that tuna tend to stay at the aFADs for 3 to 6 days and remain associated within 2 miles from the aFADs. Strong currents are the main factor for tuna departure from aFADs, though high sea surface temperatures and stormy conditions were also thought to contribute to departures. Slight currents helped tuna to aggregate at the aFADs while suitable temperature, prey and attractants enhance this aggregation. There are multiple schools segregated according to size and species present at aFADs. Catchability is higher at dawn and in the late afternoon when the tuna surface. This study is an important step towards engaging the Maldivian tuna fishers in science-based fishery management.

Introduction

The Maldives tuna fishery has existed for over a millennium [1]. This traditional livebait pole-and-line fishing technique has not changed much over the years, although fishers moved from small wooden boats that depended on wind and sail for propulsion to large (25 m) mechanized vessels [2]. In the Maldives, all tuna are caught by hook and line and all other gears such as purse seine, gillnets or trawl nets are forbidden. In the last four decades, the fishery has increased production with catches increasing from 30,000 tons in 1980 to 139,000 tons in 2017. About 75% of the catch, mainly skipjack and small yellowfin (<65cm) tuna, is caught using pole-and-line [3], the other main gear being handline.

Within the pole-and-line tuna fishery there are two distinct fleets: the artisanal (subsistence) fleet and the commercial fleet. The artisanal fleet comprises small vessels (<20m in length) conducting day trips and usually selling their catches in the neighbouring islands mainly for local consumption. There can be up to 10 fishers working on these vessels. The commercial fleet comprises of large vessels (about 30m in length) with up to 30 crew members. These large vessels are designed to cope with rough sea conditions and can stay out at sea for a few days. They have insulated holds and carry ice to keep the fish fresh. Most of these commercial pole-and-line vessels operate in the south of the Maldives but they do travel north when the fishing gets better in the north. According to the Ministry of Fisheries, Marine Resources and Agriculture (MoFMRA) in 2018 there were 785 licensed local commercial tuna fishing vessels, owned by various families or individuals living in the islands. More than 17,500 active fishers (MoFMRA statistics, 2019) work on the vessels catching tuna using pole-and-line and handline within the Maldives EEZ, about 75 miles from the coast. Tuna are caught by targetting (i) free swimming schools, (ii) logs or drifting objects, which includes drifting fish aggregating devices (dFADs), (iii) dolphin associated schools, and (iv) anchored fish aggregating devices (aFADs). Traditionally, Maldivian tuna fishers have fished at logs or drifting objects associated schools for centuries and they refer to these objects that attract tuna as ‘*oivaali*’ (a local name given to drifting objects). Thus, in the Maldives aFADs are called *‘oivaali kandhufathi’*.

The aFAD fishery in the Maldives began in 1981 [4], with experimental FADs deployed by the Ministry of Fisheries and Agriculture. Studies conducted by Anderson and Waheed [5] suggested that the expansion of the aFADs network would help further increase tuna catches in the Maldives. With the success of this fishery, the number of aFADs deployed increased from an initial 10 (in late 1980s) to 55 in 2019 (Fig 1). Currently, the aFADs in the Maldives are deployed on average about 20km from shore, in water depths of 1000 to 2800m (MoFMRA, 2019). Only pole-and-line fishing is permitted within a 3-mile radius of the aFADs.

Recent studies showed that aFADs related catches contribute to more than one third of all the tuna caught by pole-and-line in the Maldives [6]. One particularity of the aFAD array in the Maldives is the relative low density of these devices, (i.e. the large distances between neighbouring FADs). While other aFAD arrays are characterized by short distances between FADs, (e.g. 2-14 km in Mauritius [7] or 7-31 km around Oahu, Hawaii [8]), distances between neighbouring aFADs in the Maldives range from 30 to 95 km [9].

Fishers spend a lot of time observing, understanding and accumulating information on fish behaviour and ecology [10, 11, 12] and Local Ecological Knowledge (LEK) or Fishers Ecological Knowledge (FEK) has thus become very useful in situations where minimum data are available [13]. Today, the LEK approach is being utilised in various parts of the world and is regarded as an important source of information [14]. For instance, LEK has been used to study migration [15], spatial distribution [16], habitat use [17], meso-scale behaviour [18], and fine-scale behaviour of fishes [19]. LEK has been recognised to have an important role in conservation and in developing policies/regulations related to ecosystem and resource management [20]. In the Philippines, LEK has also been used to improve the understanding of tuna behaviour at dFADs [19, 21] and at aFADs[22].

Fisher observations of fish behaviour provide very useful information for fisheries researchers [23]. For more than three decades Maldivian tuna fishers have extensively used aFADs, regularly visiting the aFADs and chumming with livebait to attract tuna during pole-and-line fishing. Information from these knowledageable fishers is important to understand tuna behaviour around the aFADs and to inform local fishery management. The objective of this study is to improve knowledge of the behaviour of tuna in relation to aFADs in the Maldives, through the fisher’s local ecological knowledge. Particularly, this study focuses on: (1) the association of tuna with individual aFADs and the aFAD network; (2) departure of tuna from aFADs; and (3) seasonal variation in abundance of tuna around aFADs in the Maldives.

Materials and methods

**Study site**

The Maldives extends from about 7^o^N to 0.5^o^S stretching 822 km (Fig 1) and is 130 km at its widest. It is subjected to the seasonal monsoons. During the northeast monsoon period the wind and current is from the northeast and east. During the southwest monsoon period the wind and current is from the southwest and west. From the north up to about 2.5^o^N, the Maldives atolls are arranged in a double chain and below 2.5^o^N the atolls form a single chain and are separated with wide deep channels through which migratory fish such as tuna travel . In the south of the Maldives, the effect of the monsoon related currents is diminished and influenced by the equatorial systems [24]. About two thirds of the yearly pole-and-line catch are landed by vessels operated in the south of the Maldives (MoFMRA, 2019).

**Fisher’s interviews**

In the Maldivian tuna fishery, the captains or fishing masters (Locally called - *Keyolhu*) start their fishing career as a crew member on a pole-and-line vessel and work their way up to become a captain. They are responsible for making the decisions related to the fishing strategy – based on the information obtained from the surrounding environment by visual observations using binoculars and bird radar. In addition, the fishing strategy is also influenced by prior knowledge on the oceanographic conditions, weather and previous day’s fishing activities in the surrounding atolls. Based on this information, the captain sets the course and steers the vessel.. The captain is also responsible for taking appropriate decisions when approaching tuna schools and manoeuvring the vessel during fishing events. The deputy captain works closely with the captain and during his absence, makes all the necessary decisions.

To ensure the validity of LEK and the quality of information obtained, it is important to select participants who have the appropriate knowledge for the interviews (Johannes et al. 2000). The fishers for the interviews were selected based on their fishing experience and area of fishing. Hence captains (n=36), deputy captains (n=9) and crew members (n=9) from 36 vessels, who had a minimum of 8 years of experience on a licensed pole-and-line vessel in the Maldives were selected. For this study, the Maldives was divided into north and south area at 2.5^o^N (Fig 1). To limit potential geographical biases on the response of fishers from the north and the south of the archipelago, 34 fishers from the south and 20 fishers from the north were selected.

A questionnaire, developed to obtain fishers perceptions of the aggregations of tuna around aFADs, was not distributed to the fishers. Instead,questions were addressed through personal interviews conducted in the local language (Dhivehi) at commercial fish landing sites and fishers home ports (local islands) where their vessel is based. The questionnaire was not filled in the presence of the fishers since most fishers are reluctant to express freely when the interview becomes too formal. Interviews lasted about 30 minutes. All 54 fishers who contributed to the study provided verbal consent. No written consent was obtained, since fishers were reluctant to sign any such documents. The study was approved by Marine Research Centre of the Ministry of Fisheries Marine Resources and Agriculture, Maldives.

To ensure consistency in the interpretations of the responses, all the interviews were conducted by the same individual (who had worked with tuna fishers for the last 10 years) in a friendly atmosphere and in an informal manner during 2017 and 2018. All fishers contributed enthusiastically to the interviews. For each question, the responses of the fishers were aggregated and converted into percentages. The questionnaire consisted of 11 questions and is included in Appendix 1.

**Logbook data**

A logbook has been used by MoFMRA to obtain fishing operation related information from tuna fishers since 2010. All licensed pole-and-line fishing vessels report the mid-day position of the vessels during fishing opertations and their daily catch in the logbook. The mid-day positions of the fishing vessels reported for the two monsoons, northeast monsoon (December to March) and southwest monsoon (May to October) were aggregated and used for calculating the percentage number of trips made during each monsoon season on the east and west side of the Maldives. April and November were considered as inter-monsoon periods and not included in the analysis. Logbook data of 2016 and 2017 were used to assess the seasonal variation in fishing grounds reported by fishers during the interviews.

**Statistical analyses**

Fishers responses on the monthly variation in tuna abundance (question 1 of the questionnaire) concerned five school types (aFAD, log, dFAD, free swimming, seamount) and the two main target tuna species skipjack tuna (*Katsuwonus pelamis*) and yellowfin tuna (*Thunnus albacares*). These responses were analysed by developing a data table with 12 columns (the months) and 540 rows (the answers of the 54 fishers for the 5 school types and the 2 species skipjack and yellowfin tuna). The accumulated information was subjected to a centred Principal Component Analysis (PCA). Four between-groups PCAs [25] were then performed to compute the ratio of variability explained by (i) the position of fishers (captains, deputy captains and crew members), (ii) the origin of fishers (north and south), (iii) the different types of schools and (iv) the species (skipjack and yellowfin). Monte-Carlo tests [26] with 1000 permutations were finally conducted to assess the significance level of the between-groups analyses. The analyses were performed using the ade4 package [27] of the R statistical language [28].

Results

The responses provided by the fishers at the scale of the aFAD array and at the scale of individual aFADs are presented in Tables 1 and 2, respectively.

**Seasonal variation and tuna association with aFADs**

In the interviews, fishers globally agreed that there is a seasonal variation in abundance of tuna in the Maldives around the aFADs (Table 1). All fishers observed that during the northeast monsoon there are more tuna on the east side and during the southwest monsoon there are more tuna on the west side of the Maldives. The mid-day positions of pole-and-line fishing vessels obtained from logbooks during the northeast and southwest monsoons (Fig 3) showed a similar trend, with fishers tending to fish more on the east (45% - fishing events) than on the west (32% - fishing events) of the Maldives during the northeast monsoon and on the west (42% - fishing events) than on the east (39% - fishing events) of the Maldives during the southwest monsoon. This pattern is also observed around the aFADs. When specifically asked about the tuna abundance along the year, it was highlighted that most skipjack and yellowfin were found around aFADs during the northeast monsoon (Fig 2).

The first two axes of the PCA (Fig 4) summarized respectively 20% and 14% of the information contained in the responses of the fishers about the seasonal variability of tuna abundance. The first axis opposed positive answers (all month projected on the left side) to negative answers (no month on the right side) (Fig 4A). As responses relative to aFADs were located mainly on the left side (Fig 4D), aFADs were considered by the fishers as globally attractive all-around the year. On the right side of axis 1 were the Log Schools, Free Schools and Seamounts, considered as globally less attracting fish. The second axis identified two groups of months: September and November to February on the upper side and the other months on the lower side (Fig 4A). The grouping of responses by school type (Fig 4D) allowed to conclude that, according to the fishers, more tunas were present around dFADS during the northeast monsoon, while more tunas were present around aFADs towards the beginning and end of the southwest monsoon.

Between-groups analyses and the associated Monte-Carlo tests confirmed that the abundance of fish over the months varied significantly according to the different types of schools (18.1% of explained variability – p = 0.001). However, there was no significant difference (p = 0.133) between skipjack and yellowfin tuna. The responses of the fishers according to their position (captain, deputy captain, and crew) did not differ (p = 0.515) while responses according to their origin (from the south and north of the Maldives) differed significantly (p = 0.002) but only explained 0.6% of the variability of the data table.

**Tuna behavior at aFADs**

Almost 90% of the fishers said that when tuna are present in the aFADs array, two adjacent aFADs never had equal amounts of tuna (Table 1). Most of the fishers (64.8%) believe that there are multiple schools of tuna at the aFADs while others (27.2%) think that there is one large mixed school (Table 2). All fishers observed that there is a diel pattern in the behaviour of tuna: tuna move closer to the surface during sunrise and later afternoon, increasing their catchability at aFADs. Only very few fishers (11%) thought that there is a variation in the horizontal distance of tuna from aFADs over the day. Nearly half (40.7%) of the fishers interviewed thought that tuna stayed at the aFADs for 3 to 6 days while one third (29.6%) believed that they stay less than 3 days. Very few fishers suggested that aggregations can last for more than 10 days (but they specified that this occurs at very few aFADs during very good fishing periods). Most of the fishers (74%) suggested that tuna could be attracted to aFADs from up to 2 miles. Few (18%) suggested that it could extend to 5 miles. Some suggested that this range could be area and season specific too.

Most of the fishers (83%) believed that current was the most important factor that drives tuna aggregations (Table 2). Slight currents help to aggregate tuna at the aFADs. Almost half of the fishers (48%) thought that attractants on the aFADs also contribute to the formation of aggregations while 46% fishers agreed that presence of prey (food for tuna) is also an important factor for the aggregations to form. About one-third of the fishers identified suitable sea surface temperature and average sea condition as other important factors. Some fishers believed that less turbid waters (20%) and sharks (18%) also play a role in the formation of aggregations.

Strong currents were identified as the most important factor (85% fishers) that lead to the departure of tuna from aFADs (Table 2). Nearly half of the fishers (55.6%) thought that loss of attractants on the FADs could also result in the departure of tuna. High sea surface temperature and absence of prey were also identified as factors by 40.7% fishers. Stormy conditions such as very rough seas (37% fishers) and predators such as dolphins (29.6% fishers) cause the tuna to leave the aFADs. Few fishers (13%) thought that high turbidity could also contributed to tuna departure.

Discussion

The present study provides new insights on the spatio-temporal distribution and behavior of tuna in the Maldives through the knowledge of tuna behaviour that fishers have experienced during several years at sea. Up to date, the only study investigating the behaviour of tuna at aFADs in the Maldives consisted in an electronic tagging experiment on skipjack and yellowfin tuna conducted in the eastern part of the Maldives, where 8 FADs were instrumented with acoustic receivers and 61 fish tagged [9]. Such experiments are expensive and logistically challenging (cost of the equipment, chartering vessels). Therefore, it is difficult to collect such data across different seasons and regions of the aFAD array. The use of the fisher’s knowledge provided a good alternative method to collect information on tuna behaviour in the Maldives.

In the Maldives tuna fishers do not use sonars or echosounders for locating or observing tuna schools during their pole-and-line operation and all 54 fishers who took part in the interviews had no knowledge of the scientific publications related to tuna behaviour around FADs. Hence fisher’s perceptions of tuna behaviour were completely based on what they had observed at the surface of the water during their fishing events. These experienced fishers, over the years, have accumulated vast amounts of knowledge on various aspects of the tuna behaviour. All of them were enthusiastic to share their knowledge and experiences.

**Seasonal variation and tuna association with aFADs**

The seasonal variation in abundance of tuna in the Maldives observed by the fishers (increase of abundance on the east of the Maldives during northeast monsoon and more tuna found on the west of the Maldives during southwest monsoon) and confirmed through logbook data, is similar to what was reported by Adam and Anderson [29]. But this pattern is not so obvious in the south of the Maldives. Although the northeast monsoon is shorter than the southwest monsoon, in general, there are high catches observed during the northeast monsoon (MoFMRA logbook data). This could be due to severe weather conditions experienced during the southwest monsoon, making it difficult for fishers to conduct their fishing operations. Fishers encounter more natural floating objects (log) associated schools during the northeast monsoon (Fig 2) and believe that drifting objects associated tuna and free-swimming schools of tuna move towards the Maldives with the monsoon currents. Similar observations were made by Adam and Anderson [29], with a pattern that could correspond to tuna entering the Maldives from the north (on the east side) at the beginning of the northeast monsoon and tuna entering from the south (on the west side) towards the beginning of the southwest monsoon.

Almost all the fishers agreed that they have never observed equal amounts of tuna at two adjacent aFADs but a few said that, when tuna was very abundant around the atolls they had, on few occasions, seen similar aggregations at two adjacent aFADs. The origin of this question relates to the fact that for social species (e.g. tuna), the competition of two attracting devices could lead to one of the devices aggregating most of the local population [30]. Robert et al., [31] designed an experiment with aFADs in the Seychelles to test this hypothesis on tuna and showed that tuna generally aggregate to one of the two close FADs. In their experiment, however, FADs were only 5 km apart. In the Maldives, two adjacent FADs are 30-95 km apart. According to fishers, although FADs are quite far from each other, the selection of only one FAD seems to be a common observation. New experiments, using echosounder buoys [31, 32] for instance, attached to aFADs in the Maldives, could further investigate this result.

Most fishers believe that multiple schools of tuna, segregated by species and size, form the aggregation around aFADs. These fishers observed that during fishing operations around aFADs, when several vessels fish simultaneously, some vessels caught only small size fish while other vessel caught bigger fish. The same pattern was observed by Moreno et al., [19] where skippers of purse seines also considered that several tuna schools form the aggregations around dFADs, segregated by species and sizes. Macusi et al., [22] also reported a similar pattern for tuna at aFADs in the Philippines. Some fishers, however, believe that there is one large school where different species and sizes mix. It is very likely that both situations occur at aFADs, depending on the local biomass and conditions.

All fishers observed a diel pattern in the tuna behaviour, with fish moving closer to the surface more aggressively as they feed on the livebait during sunrise and late afternoon, increasing their catchability at the aFADs at dawn and the very early hours of the morning. A study by Macusi et al., [22] and acoustic telemetry at FADs; e.g. [33, 34] also showed that fish tend to aggregate near the surface at the FAD during the night/early morning. Few fishers thought there was some variation in the horizontal positioning of tuna at aFADs during the day. Schaefer and Fuller during their ultrasonic telemetry experiments around dFADs in 2013 [33] observed that all three species of tunas (skipjack, yellowfin and bigeye tuna) tend to move away from the FAD and return to it after few hours. However, it is possible that the fishers in the Maldives, that fish at the aFADs only during the daytime, do not experience such horizontal movements.

The time tuna spend at FADs is considered as a key parameter to characterize its associative behaviour. Nearly half of the fishers thought that tuna stay at aFADs for 3 to 6 days. Only very few fishers reported that they had observed aggregations that lasted for more than 10 days at few aFADs and only during very good fishing periods. In the study by Macusi et al., [22] in the Philippines, nearly half of the fishers interviewed believed that tuna stays at aFADs for 2 weeks, which is considerably longer than what is reported by Maldivian fishers. Several acoustic tagging studies were designed to document the time individual tuna spend associated with aFADs and dFADs, including a study in the Maldives, which could directly be compared to our study.

During the acoustic tagging study conducted in 2009 in the Maldives [9] the average continuous residence time (CRT) for tuna varied from 0.7 days for yellowfin to 3.5 days for skipjack tuna. A similar study conducted in Mauritius [7] showed that the average CRT was between 2.5 and 9.6 days for skipjack and yellowfin tuna respectively. Although the CRT observed for yellowfin tuna at the aFADs in the Maldives [9] was much shorter (average 0.66 days) than any other study on the same species around aFADs [7, 8, 35, 36, 37] the observations made by fishers (3 to 6 days) were within the range of CRT values observed in most of the studies. Acoustic tagging provides measures of the residence times of individual tuna at aFADs, while fishers mainly collect their knowledge on the duration of tuna aggregations at FADs. The residency of individuals and aggregations could be different, as aggregations could result from the turn-over of individuals that is composed by them: some fish arrive while other leave the FAD, but the aggregation can be maintained. Combining experiments with acoustic tagging and echosounder buoys would allow to link individual and collective behaviour and would facilitate the comparison with fishers knowledge.

Most of the fishers suggested that tuna could be attracted to aFADs from up to 2 miles (about 3.7 km) while some fishers suggested that the attraction distance varies depending on the area and the season. Skippers of tuna purse seiners considered that tuna could be attracted towards dFADs from 0 – 5 nautical miles (0 – 9 km) [19, 21]. Using data collected from active acoustic tracking of tuna at aFADs in the Indian and Pacific Oceans [38, 39, 40, 41] found that the orientation distances ranged from 4 to 19 km, with a mode at about 10 km. This was similar to the estimates of the skippers of the purse seiners but the attraction distance suggested by Maldivian fishers are lower. This could be because the fishers beyond 2 miles distance the aFADs are not visible to the fishers and though tuna are attracted to the aFADs, fishers do not associate (consider attracted) with aFADs. Active tracking of tuna at aFADs in the Maldives would document the attraction distance more precisely, but it is noteworthy that all studies, including ours, tend to suggest that tuna could be attracted from a few kilometers to aFADs.

**Formation of tuna aggregations and departure of tuna from aFADs**

Most fishers believed that more than one single factor contributes towards the formation of tuna aggregations at the aFADs and its dissolution. A large majority agreed that the currents had the highest impact on the aggregations around aFADs. Skippers of large tropical tuna purse seiners also reported that strong currents, changes in temperature and violent sea conditions can have a strong impact on the tuna aggregations at FADs [19]. Macusi et al. [22] also identified changes in sea currents as one of the main reasons for tuna departure from aFADs in the Philippines. Several other studies have also acknowledged that the duration for which tuna stayed at the FADs and their departure could be linked to the oceanographic condition around the FADs [39, 42, 43].

About half the fishers believed that the presence or absence of prey species affect aggregations. These fishers believed that tuna feed on various other smaller marine organisms at the vicinity of the aFADs and the abundance of tuna tends to follow the prey abundance. Several studies have suggested that prey availability around the FADs affects the duration of the aggregations [35, 44, 45]. In the pole-and-line tuna fishery of the Maldives, fishers almost daily chum large quantities of livebait at the aFADs and some fishers suggested that this could encourage the tuna to remain at the aFADs longer. Most fishers did not believe that large school size leads to tuna departures from the aFADs. This is also similar to the observations made by the purse seine fishers [21].

Some fishers also suggested that large predators such as sharks and dolphins can affect the behaviour of tuna. Fishers consider that sharks associated with aFADs could somehow help maintain tuna aggregations at aFADs. This striking information has never been documented in any other scientific publications. Forget et al., [34] and Filmalter et al., [46], using acoustic tracking, showed that silky sharks made excursions away from a dFAD, being closely associated with a school of tuna. While these studies indicate that silky sharks and tunas tend to exhibit similar associative patterns, they do not allow to investigate whether sharks play a role in the associative behaviour of tuna to floating objects. Fishers also observed that continuous chasing of tuna by predators negatively affected catchability of tuna. The same effect of marine mammals on the departure of tuna from FADs was reported both by fishers fishing on aFADs in the Philippines [22] and by skippers of purse seiners fishing on dFADs [21]. This convergence of results tends to show a general cause-effect of mammals on the residency of tuna at FADs.

Maldivian fishers believe that attractants on aFADs also influence the tuna aggregations. The aFADs deployed in the Maldives have a set of small floats weaved together that provides a shade and protection for small fish associated with the aFAD. Sometimes fishers attach pieces of thick ropes or occasionally, pieces of nets that they have recovered from dFADs to serve as attractants. Tuna fishers regularly check on the attractants attached to the aFADs and if the attractants are missing from an aFAD, they inform MoFMRA to attach new attractants. They believe these attractants provide shelter to the small fish that help to form tuna aggregations.

The Maldives aFAD fishery is one of the important tuna fisheries in the Indian Ocean in terms of annual catches and its role in providing food security for the local people. This fishery has existed for several decades and its sustainability shows the importance of good management strategies practiced by the fishers over the years. With increase in demand for tuna, both locally and internationally, it is important to ensure the sustainability of the fishery for future generations. Thus, understanding the behaviour of tuna around aFADs in a country like the Maldives is key for both the local management of the national fisheries and the regional management conducted at the scale of the Indian Ocean. The methodology used in this paper (LEK) is particularly important as it contributes to engage local fishers to science for management. It is through such participation in research activities that fishers fully realize the importance of fisheries research and the importance of their contributions to the overall management of the tuna fisheries in the Maldives. It is hoped that this study will pave the way to engage fishers in resource management.

References

1. Adam MS. “Population Dynamics and Assessment of Skipjack Tuna (*Katsuwonus pelamis*) in the Maldives.” Unpublished Doctoral Thesis, Imperial College of Science and Technology. 1999. – 303 pages.
2. Adam MS, Anderson RC, Hafiz A. Tuna Maldivian tuna fishery. 2003. IOTC Proceedings no. 6 pages 202-220. WPTT – 03 – 23
3. Ahusan M, Adam MS, Ziyad A, Shifaz A, Shimal M, Jauharee AR. Maldives National Report to the Scientific Committee of the Indian Ocean Tuna Commission. 2018.
4. Naeem A, Latheefa A. Biosocioeconomic assessment of the effects of fish aggregating devices in the tuna fishery in the Maldives. Bay of Bengal Programme, Madras. 1994; WP/ RAS/91/006.
5. Anderson RC, Waheed A. Exploratory fishing for large pelagic species in the Maldives. Bay of Bengal Program, Fisheries Development India. 1990. BOBP/REP/46.
6. Miller KI, Nadheeh I, Jauharee AR, Anderson RC, Adam MS. Bycatch in the Maldivian pole-and- line tuna fishery. PLoS One. 2017; 12(5)
7. Rodriguez-Tress P, Capello M, Forget F, Soria M, Beeharry SP, Dussooa N, Dagorn L. Associative behavior of yellowfin *Thunnus albacares*, skipjack *Katsuwonus pelamis*, and bigeye tuna *T. obesus* at anchored fish aggregating devices (FADs) off the coast of Mauritius. Mar Ecol Prog Ser. 2017; Vol. 570: 213–222.
8. Dagorn L, Holland KN, and Itano DG. Behaviour of yellowfin (*Thunnus albacares*) and bigeye (*Thunnus obesus*) tuna in a network of fish aggregating devices (FADs). Mar. Biol. 2007;151-595-606.
9. Govinden R, Jauharee AR, Filmalter J, Forget F, Soria M, Adam MS, Dagorn L. Movement behavior of skipjack (*Katsuwonus pelamis*) and yellowfin (*Thunnus albacares*) tuna at anchored fish aggregating devices (FADs) in the Maldives, investigated by acoustic telemetry. Aquat Living Resources. 2013;26, 69-77
10. Baird IG, Flaherty MS. Mekong river ﬁsh conservation zones in southern Laos: assessing eﬀectiveness using local ecological knowledge. Environ. Manag. 2005; 36, 439–454.
11. Johannes RE, and Neis B. “The value of anecdote,” in Fishers’ Knowledge in Fisheries Science and Management, eds Haggan N, Neis B, and Baird IG. Paris: UNESCO Publishing. 2007; 35–58.
12. Lavides MN, Polunin NVC, Stead SM, Tabaranza DG, Comeros MT, and Dongallo JR. Finﬁsh disappearances around Bohol, Philippines inferred from traditional ecological knowledge. Environ. Conserv. 2010; 36, 235–244.
13. Silvano RAM, Valbo-Jorgensen J. Beyond fishermen’s tales: contributions of fishers; local knowledge to fish ecology and fisheries management. Environ Dev Sustain. 2008; 10:657–675
14. Ruddle K, Davis A. Local Ecological Knowledge (LEK) in Interdisciplinary Research and Application: a Critical Review. Asian Fisheries Science. 2013; 26: 79-100.
15. Valbo-Jorgensen J, Poulsen AF. Using local knowledge as a research tool in the study of river fish biology: experiences from the Mekong. Environ. Dev. Sustain. 2001; 2: 253–276
16. Poizat G, Baran E. Fishermen’s knowledge as background information in tropical fish ecology: a quantitative comparison with fish sampling results. Environmental Biology of Fishes. 1997; 50: 435–449.
17. Silvano RAM, Begossi A. Fishermen’s local ecological knowledge on southeastern Brazilian coastal fishers: Contributions to research, conservation, and management. Neotropical Ichthyology. 2012; 10(1), 133-147.
18. Mackinson S. Integrating local and scientiﬁc knowledge: an example in ﬁsheries science. Environ. Manage. 2001; 27, 533–545.
19. Moreno G, Dagorn L, Sancho G, Itano D. Fish behaviour from fishers’ knowledge: the case study of tropical tuna around drifting fish aggregating devices (DFADs). Fish. Aquat. Sci. 2007. 64: 1517
20. Rajamani L, Marsh H. Using parallel regional-and local-scale initiatives to inform conservation management of rare wildlife: a case study of Dugong in Sabah, Malaysia. Endanger. Species Res. 2010; 13,17–23.
21. Moreno G, Dagorn L, Sancho G, Garcia D, Itano D. Using local ecological knowledge (LEK) to provide insight on the tuna purse seine fleets of the Indian Ocean useful for management. Aquat. Living Resour. 2007; 20: 367-376
22. Macusi ED, Abreo NAS, Babaran RP. Local Ecological Knowledge (LEK) on Fish Behaviour Around Anchored FADs: the Case of Tuna Purse Seine and Ring net Fishers from Southern Philippines. Front. Mar. Sci. 2017; 4:188.
23. Johannes RE, Freeman MMR, Hamilton RJ. Ignore fishers’ knowledge and miss the boat. Fish Fish. 2000; 1(3): 257–271.
24. Anderson RC. Observations of cetaceans in the Maldives, 1990-2002. J. Cetacean Res. Manage. 2000; 7(2):119–135.
25. Dolédec S, Chessel D. Rythmes saisonniers et composantes stationnelles en milieu aquatique I- Description d'un plan d'observations complet par projection de variables. Acta Oecologica, Oecologia Generalis. 1987; 8, 3, 403–426.
26. Romesburg H C. Exploring, confirming and randomization tests. Computers and Geosciences. 1985; 11, 19–37.
27. Dray S, Dufour A. The ade4 Package: Implementing the Duality Diagram for Ecologists. Journal of Statistical Software. 2007; 22(4), 1-20. doi: 10.18637/jss.v022.i04 (URL: <http://doi.org/10.18637/jss.v022.i04>).
28. R Core Team. R: A language and environment for statistical computing. R Foundation for Statistical Computing, Vienna, Austria. 2019. URL <https://www.R-project.org/>
29. Adam MS and Anderson RC. Marine Research Bulletin. 1998; Volume 3.
30. Sempo G, Dagorn L, Robert M, Deneubourg J. Impact of increasing deployment of artificial floating objets on the spatial distribution of social fish species. Journal of applied ecology. 2013; 50, 1081–1092.
31. Robert M, Dagorn L, Filmalter JD, Deneubourg JL, Itano D, Holland K. Intra-individual behavioural variability displayed by tuna at ﬁsh aggregating devices (FADs). Mar. Ecol. Prog. Ser. 2013; 484, 239–247.
32. Lopez J, Moreno G, Ibaibarriage L, Dagorn L. Diel behaviour of tuna and non-tuna species at drifting fish aggregating devices (DFADs) in the Western Indian Ocean, determined by fishers’ echo-sounder buoys. Mar. Biol. 2017; 164:44
33. Schaefer KM, Fuller DW. Simultaneous behaviour of skipjack (Katsuwonus pelamis), bigeye (Thunnus obesus), and yellowfin (T. albacares) tunas, within large multi-species aggregations associated with drifting fish aggregating devices (FADs) in the equatorial eastern Pacific Ocean. Marine Biology. 2013; 160:3005-3014.
34. Forget FG, Capello M, Filmalter JD, Govinden R, Soria M, Cowley PD, Dagorn L. [Behaviour and vulnerability of target and non-target species at drifting fish aggregating devices (FADs) in the tropical tuna purse seine fishery determined by acoustic telemetry](https://www.nrcresearchpress.com/doi/abs/10.1139/cjfas-2014-0458). Canadian Journal of Fisheries and Aquatic Sciences, 2015; Vol. 72, No. 9: pp. 1398-1405.
35. Ohta I. Kakuma S. Periodic behaviourand residence time of yellowﬁn and bigeye tuna associated with ﬁsh aggregating devices around Okinawa Islands, as identiﬁed with automated listening stations. Mar. Biol. 2005; 146: 581–594
36. Mitsunaga Y, Endo C, Anraku K, Selorio CM, Babaran RP. Association of early juvenile tuna Thunnus albacores with a network of payaos in the Philippines. Fish. Sci. 2012; 78, 15–22.
37. Robert M, Dagorn L, Deneubourg JL, Itano D, Holland K. Size-dependent behaviour of tuna in an array of ﬁsh aggregating devices (FADs). Mar. Biol. 2012; 159, 907–914.
38. Holland KN, Brill RW, Chang RKC. Horizontal and vertical movements of yellowﬁn and bigeye tuna associated with ﬁsh aggregating devices. Fish Bull (Wash DC). 1990; 88:493–507
39. Brill RW, Block BA, Boggs CH, Bigelow KA, Freund EV, Marcinek DJ. Horizontal movements and depth distribution of large adult yellowﬁn tuna (Thunnus albacares) near the Hawaiian Islands, recorded using ultrasonic telemetry: implications for the physiological ecology of pelagic ﬁshes. Mar Biol. 1999; 133:395–408
40. Dagorn L, Bach P, Josse E. Movement patterns of large bigeye tuna (Thunnus obesus) in the open ocean, determined using ultrasonic telemetry. Marine Biology. 2000; 136: 361-371
41. Girard C, Benhamou S, Dagorn L. FAD: Fish aggregating device or fish attracting device? A new analysis of yellowfin tuna movements around floating objects. Animal Behaviour. 2004; 67, 319-326.
42. Cayré P, Marsac F. Modelling the yellowfin tuna (Thunnus albacares) vertical distribution using sonic tagging results and local environmental parameters. Aquat Living Resour. 1993; 6: 1−14.
43. Schaefer KM, Fuller DW. Vertical movements, behavior, and habitat of bigeye tuna (Thunnus obesus) in the equatorial eastern Pacific Ocean, ascertained from archival tag data. Mar Biol. 2010; 157: 2625−2642
44. Musyl MK, Brill RW, Boggs CH, Curran DS, Kazama TK, Seki MP. Vertical movements of bigeye tuna (Thunnus obesus) associated with islands, buoys, and seamounts near the main Hawaiian Islands from archival tagging data. Fish Oceanogr. 2003; 12: 152−169
45. Graham BS, Grubbs D, Holland K, Popp BN. A rapid ontogenetic shift in the diet of juvenile yellowfin tuna from Hawaii. Mar Biol. 2007; 150: 647−658
46. Filmalter JD, Cowley P, Forget F, Dagorn L. Fine-scale 3-dimensional movement behaviour of silky sharks Carcharhinus falciformis associated with fish aggregating devices (FADs). Mar Ecol Prog Ser. 2015; 539: 207-223

**Appendix**

Local Ecological Knowledge – Fisher interview questionnaire

1. During which season (monsoon) are there more fish (any size) around different types of schools?

2. Is there any variation in abundance of fish around aFADs on the east and west of Maldives?
3. Are there any aFADs that generally attract less fish?
4. Are there any aFADs that generally attract more fish?
5. When aFADs are close by (adjacent) do more fish appear on one aFAD than other or are they equal?
6. When a FAD aggregates fish how many days is the aggregation around?

*Options provided:* ***<3days, 3 to 6 days, 7 to 10 days, >10days***

7. Which are the most important factors that help the formation of fish aggregation at aFADs?

*Options provided:* ***current, temperature, turbidity, presence of prey, attractants attached, sea state.***

8. Which are the most important factors that explains why fish leaves the aFADs?

*Options provided:* ***current, temperature, turbidity, absence of prey, absence of attractants, stormy seas, presence of large predators, large size of the aggregations.***

9. What is the distance that you consider that the fish is still associated to the aFAD?

*Options provided:* ***0 to 2 miles, 0 to 5 miles, >5 miles***

10. Does time of the day influence the behavior of tuna at the aFADs?
11. Do you think there are multiple schools of fish at the aFADs?

**Fig 1. The aFAD network outside Maldives atolls and the monsoon related currents**

**Fig 2. Fishers’ response on the seasonal variation in abundance of two tuna species at five types of schools in the Maldives. April and November are inter-monsoon periods.**

**Fig 3. Mid-day position of pole-line-fishing vessels from logbook data for the NE and the SW monsoon periods.**

axis 2

axis 1

A

E

D

B

C

**Fig 4. Results of the PCA of the answers of the 54 fishers for the 5 types of schools and the two species (skipjack and yellowfin tuna) per month.**

**On the first two axes, projection of the 12 months (A) and of the 540 rows grouped by origin of the fisher (B), by position of the fisher (C), by type of school (D) and by species (E). The value of d gives the scale of the grid.**

**Table 1: Fishers’ response to seasonal variation and association behaviour of tuna at aFADs, at the scale of the aFAD array.**

| Questions | Percentage (%) | |
| --- | --- | --- |
| There is seasonal variation in abundance/size of tuna around aFADs on the east and west of Maldives | Yes  94.4 | No  5.6 |
| There are more fish at aFADs on the east side of the Maldives during northeast monsoon | Yes  100 | No  0.0 |
| There are more fish at aFADs on the west side of the Maldives during southwest monsoon | Yes  100 | No  0.0 |
| When fish are present in the aFAD array – two adjacent aFADs do not have same amount of tuna | Yes  88.9 | No  11.1 |
| There are aFADs that always attract less tuna | Yes  79.6 | No  20.4 |
| There are aFADs that always attract more tuna | Yes  87.0 | No  13.0 |

**Table 2: Fishers’ responses related to formation of aggregation at aFADs, at the scale of individual aFADs.**

| Question | Percentage (%) | |
| --- | --- | --- |
| There are multiple schools at aFADs and are segregated according to fish size and species | Yes  64.8 | No  27.2 |
| Time of the day influence the behaviour of tuna at the aFADs  Horizontal distance from aFAD  Vertical distance from aFAD  Catchability | Yes  11.1  100  100 | Not sure  88.9  0.0  0.0 |
| Number of days that tuna stays at aFADs  Less than 3 days  3 to 6 days  7 to 10 days  More than 10 days | 29.6  40.7  20.4  9.3 | |
| Distance fishers consider that the tuna is attracted to the aFAD  0 to 2 miles  0 to 5 miles  > 5 miles | 74.1  18.5  7.4 | |
| Reasons for tuna to aggregate at aFADs?  Suitable current (not strong/ not very weak)  Suitable temperature  Less turbid  Presence of prey  Presence of sharks  Attractants present  Sea state (average) | Yes  83.3  37.0  20.4  46.3  18.5  48.1  29.6 | No  16.7  63.0  79.6  53.7  81.5  51.9  70.4 |
| Reasons for tuna to leave the aFADs?  Strong current  High sea surface temperature  Turbidity (very high)  Absence of prey  Presence of predators/mammals  Attractants absent  Storms / very rough sea condition  Large size of aggregations | Yes  85.2  40.7  13.0  40.7  29.6  55.6  37.0  24.1 | No  14.8  59.3  87.0  59.3  70.4  44.4  63.0  75.9 |
